# A Complicated Case of Catatonia

**DOI:** 10.1192/j.eurpsy.2022.1217

**Published:** 2022-09-01

**Authors:** A. Makela

**Affiliations:** Harvard Medical School at Cambridge Health Alliance, Department Of Psychiatry, Cambridge, United States of America

**Keywords:** Bush Francis, multiple sclerosis, inflammation, Catatonia

## Abstract

**Introduction:**

Many different causes of catatonia are well-documented in medicine. Modern understanding of catatonia has evolved in the last 100 years with the suggestion that there is a root cause in neuroinflammation. This is a case report of a young woman who presented to the emergency department with altered mental status, found to have catatonia responsive to lorazepam, with the underlying etiology being a diagnosis of multiple sclerosis.

**Objectives:**

A case-based approach is used to support the following learning objectives: - Review the diagnostic criteria for catatonia - Distinguish between simple and malignant catatonia - Review the Bush-Francis Scale - Review available treatment

**Methods:**

Mother brings 24-year-old woman into the hospital for altered mental status and changes in behavior including staring spells, periods of withdrawal, refusal to eat, lack of purposeful movement, apraxia, and mutism that worsened 24 hours prior to presentation.

**Results:**

The patient was afebile with negative covid-19 test. Recent diagnosis of Bell’s palsy treated with antivirals and oral steroird, which terminated just prior to presentation. Additionally, patient had outpatient treatment for vertigo. Lumbar puncture was negative for an infectious process. MRI revealed multiple stable white matter lesions in the periventricular, pontine, and subcortical regions, some oriented perpendicular to the corpus callosum.

**Conclusions:**

This case of a 24-year-old woman with catatonia brought an opportunity to retrospectively review a case in detail in order to feature learning objectives that review very important considerations in the evaluation, differential diagnosis, symptom tracking, and treatment of catatonia. The future of research in catatonia is bright and diverse.

**Disclosure:**

No significant relationships.

